# Post-Work Recovery from Fatigue and Sleep Episodes among Nurses Who Are Engaged in 16-Hour Night Shifts: A Prospective Observational Study

**DOI:** 10.3390/healthcare10061089

**Published:** 2022-06-11

**Authors:** Issei Konya, Kazuhiro Watanabe, Inaho Shishido, Naotaka Sugimura, Yuta Matsushita, Shinya Yamaguchi, Rika Yano

**Affiliations:** 1 Graduate School of Health Sciences, Hokkaido University, Sapporo 060-0812, Japan; ik-0v0-ik628@eis.hokudai.ac.jp (I.K.); kazu-watanabe@eis.hokudai.ac.jp (K.W.); naotaka-s@eis.hokudai.ac.jp (N.S.); s_yamaguchi@eis.hokudai.ac.jp (S.Y.); 2 Research Fellow of Japan Society for the Promotion of Science, Tokyo 102-0083, Japan; 3 Faculty of Health Sciences, Hokkaido University, Sapporo 060-0812, Japan; inaho_s@hs.hokudai.ac.jp; 4 Graduate School of Education, Hokkaido University, Sapporo 060-0811, Japan; matsushita.yuta.o6@elms.hokudai.ac.jp

**Keywords:** age, fatigue, nurses, shift work, sleep, recovery

## Abstract

Poor recovery from fatigue among shift-working nurses can cause a transition from acute to chronic fatigue. We aimed to clarify the relationship between nurses’ recovery from fatigue and sleep episodes after 16 h night shifts while considering age. This prospective study included 62 nurses who worked 16 h night shifts. Fatigue was assessed by a questionnaire before, during, and after the night shift, and the morning following the night shift. Sleep episodes were continuously measured using a wearable device. We performed a hierarchical cluster analysis of multivariate sleep parameters in first and main sleep episodes after night shifts. A linear mixed model was used to estimate the difference between clusters in recovery from fatigue after the night shift, considering age. The participants were classified into a high sleep quality group (HSQG) and low sleep quality group (LSQG) in sleep episodes after the night shift. There was a significant main effect of clusters, and HSQG was significantly more effective than LSQG in recovering from fatigue. However, no main effects of age or interaction were observed. The quality of first and main sleep episodes at home was associated with recovery from the night shift to the next day, regardless of age.

## 1. Introduction

Shift work in nursing is essential for providing a 24/7 health care system for the community. However, long shifts can disrupt circadian rhythms, impose high work demands, and cause acute fatigue [1]. Acute fatigue is a temporary lack of energy due to work, and if it is not relieved by appropriate rest, it can lead to decreased performance [1,2]. One of the most effective measures to relieve acute fatigue is to take a nap during the night shift [3,4]. In addition, although “recovery” during non-working time plays an important role in the fatigue caused by work, low levels of recovery can cause a transition from acute fatigue to chronic fatigue [1]. Chronic fatigue is associated with various health risks, including musculoskeletal disorders [4], sleep disturbances [3], burnout [5], poor well-being [6], anxiety, and depression [7], as well as a reduction in the quality of nursing care and a threat to patient safety [2]. Therefore, it is desirable to promote sufficient recovery from fatigue through appropriate sleep after night shifts to prevent the transition from acute to chronic fatigue [1,8].

In Japan, the two-shift system is becoming more common, replacing the traditional three-shift system; 52.5% of wards with a two-shift system have introduced a 16 h night shift [9]. Notably, a 16 h night shift, which is longer than the 12 h shifts prevalent in Europe and the United States, is expected to cause excessive acute fatigue among nurses [10]. Thus, to ensure nurses’ health and patient safety, it is necessary to examine the relationship between fatigue, napping, and sleep among nurses working long night shifts.

Napping during night shifts is expected to compensate for sleep deprivation and promote the alignment of circadian rhythms [3]. Previous studies have reported that napping at night prevents increased body mass index, hypertension, cardiovascular disease, poor performance, daytime sleepiness [3,11], and fatigue [12], and promotes recovery after work [13]. Moreover, sleeping at home after a night shift is essential to prevent the transition from acute to chronic fatigue. Since sleeping at home is less affected by the work environment than by napping during the night shift, it is easier for individuals to devise their sleep habits. The guidelines on night shift for nurses, published by the Japanese Nursing Association, recommend that nurses should obtain about two hours of a first sleep episode as early as possible after their night work, live a normal life during the day so as not to disrupt their circadian rhythms, and go to bed as usual or a little earlier at night [14]. Contrary to these recommendations, a study showed that the percentage of nurses who slept in the morning after a 16 h night shift was low, especially among those in their 20s [12]. This study also showed that nurses who had an early sleep phase after their night shift (taking a nap in the morning and going to bed early at night) had significantly lower cumulative fatigue than nurses who had a late sleep phase (taking a nap in the afternoon and going to bed late at night) [12]. However, it remains unclear how sleep episodes such as the first and main sleep after a night shift affects nurses’ degree of recovery from fatigue. Moreover, few studies have longitudinally investigated the relationship between fatigue and sleep episodes during non-working hours after night shifts [1].

In addition, the effect of age should be carefully considered when examining the abovementioned topics. Gifkins et al. [1] also reported age as one of the factors impeding recovery from fatigue at home among shift-working nurses. Most nurses are women and, as such, are prone to a rapid decline in estrogen and progesterone levels, beginning in their 40s, during menopausal transition [15,16]. These hormonal changes are associated with fatigue and sleep disturbances such as poor sleep quality, insomnia, fragmented sleep, and nighttime wakefulness [17,18,19]. This age effect is the most significant confounding factor related to nurses’ fatigue recovery and sleep. Without an accurate understanding of this confounding factor, it is impossible to plan appropriate measures for nurses’ health.

Therefore, this study aimed to clarify the relationship between recovery from fatigue and sleep episodes (namely first and main sleep episodes) after 16 h night shifts, while also considering the effect of age on these nurses. This study provides relevant data that can be used to provide suggestions for future policies aimed at reducing fatigue among nurses who work long shifts.

## 2. Materials and Methods

### 2.1. Operational Terms

**Figure 1:**
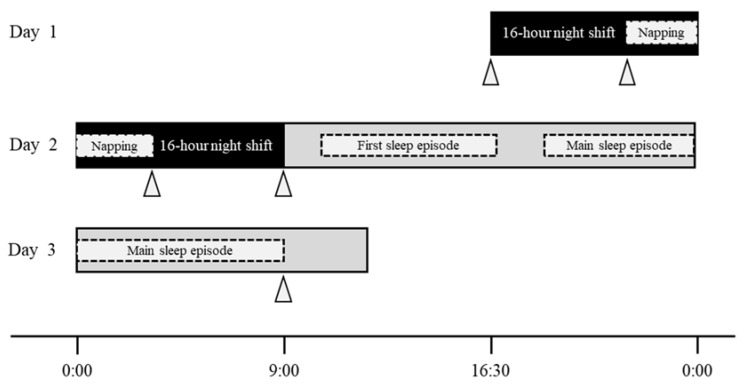
Definition of “16 h night shift” and graphic summary of study protocol. ***Notes****:* the dotted areas indicate that there was individuality among the participants; the gray areas after the night shift indicate non-working time; the participants were asked to wear the wearable device (MTN-220) for assessing participants’ sleep/ activity from before the night shift (Day 1) to the morning of the next day after the night shift (Day 3); triangles indicate the measurement time of fatigue by the “Jikaku-sho shirabe.”

- **16 h night shift**: a night shift from 16:30 to 09:00 the next day in a two-shift system (Figure 1).
- **Night-shift napping**: a short sleep taken during the night shift.
- **First sleep episode**: the first sleep episode after the night shift, excluding the main sleep episode.
- **Main sleep episode**: the longest sleep episode between the period after a night shift and the morning of the day after this shift.

### 2.2. Study Design and Participants

This prospective observational study was conducted in a general hospital with over 200 beds in northern Japan. We observed one night shift (three days) per nurse between December 2019 and March 2020 (Figure 1). In total, 151 nurses belonged to a wide range of wards, including internal medicine wards such as cardiology and gastroenterology, surgical wards such as neurosurgery, ophthalmology, and plastic surgery, and rehabilitation wards for convalescent period patients. Of these, registered nurses in their 20 s to 40 s who worked in a two-shift system, including 16 h night shifts (16:30–09:00), were the participants. The exclusion criteria were as follows: (1) less than one year of experience as a nurse, (2) regular use of sleeping pills, (3) pregnancy, and (4) nursing managers and administrators. The eligible nurses were classified into three age categories: 20–29 years (20 s), 30–39 years (30 s), and 40–49 years (40 s). Finally, 66 nurses who met the eligibility criteria participated, excluding four that declined to take part in the study.

The study objectives, methods, and anonymity-related procedures were explained verbally and in writing to those who met the eligibility criteria and expressed interest in participating. All participants provided written and verbal informed consent for publication before participating in this study. This study was approved by the Ethical Review Board of the university to which the authors are affiliated and the participating facility (reference No. 19–65). It was carried out according to the Declaration of Helsinki.

### 2.3. Outcome Measures

#### 2.3.1. Demographic Data

Participants were asked to provide the following individual data: age, sex, body mass index, marital status, having a child, years of nursing experience, frequency of drinking and caffeine intake, and habit of taking a nap before the night shift. The questionnaire was administered at the beginning of the study.

#### 2.3.2. Fatigue

Work-related fatigue was measured by the 25-item “Jikaku-sho shirabe,” which was developed by the Working Group for Occupational Fatigue, as part of the Japan Society for Occupational Health [20]. This questionnaire has also been used as a scale to measure fatigue in nurses [12]. It comprises five factors: feeling of drowsiness, instability, uneasiness, local pain or dullness, and eyestrain. For each item, the participants were asked to answer using the following scale: completely disagree (1 point), scarcely agree (2 points), slightly agree (3 points), considerably agree (4 points), and strongly agree (5 points). The total score for each factor and the overall total score were used, with a higher score indicating more fatigue. The measurement points were set as follows: before the night shift (Day 1), before and after napping during the night shift and after the night shift (Day 2), and in the morning of the day after the night shift (Day 3, Figure 1).

#### 2.3.3. Sleep and Napping

##### Activity/Sleep

The already-validated MTN-220 (ACOS CO., LTD., Iida, Japan) was used for objectively assessing participants’ activity/sleep [21]. This is a small, light, and round wearable device (diameter: 27.0 mm; thickness: 9.1 mm; weight: 9.0 g) that records the amount of activity by using an internal three-axis accelerometer. Data on the amount of activity and participants’ postures were collected every two minutes. The participants were asked to wear the device on the front side of their trunks, clipping it to the edge of their pants/trousers, from before the night shift (Day 1) to the morning of the day after the night shift (Day 3, Figure 1). 

Data were obtained from the devices through a near-field communication interface (PaSoRi, RC-S380, Sony Corporation, Tokyo, Japan) using the SleepSignAct (SSA) software (Kissei Comtec, Matsumoto, Japan). To detect sleep/awake states based on data from the devices, the default settings of SSA were employed, in which sleep detection followed a previously reported algorithm [22].

The cumulative steps during the night shift were extracted as the total number of steps taken from 17:00 (Day 1) on the first day of the night shift to 9:00 (Day 2) the next day.

##### Questionnaire for Night-Shift Napping and Sleep Diary for after the Night Shift

Participants were asked to record subjective evaluations of their night-shift napping using a questionnaire, comprising items on whether they did or did not nap, their rest time, and napping time. Moreover, participants were asked to use a sleep diary to record their activities, sleep time, and the times they removed the MTN-220 during the period from after the night shift to the morning of the following day.

##### Analytical Methods for Sleeping and Napping Data

Sleep parameters were calculated considering both objective data (analyzed by SSA) and subjective data for napping and sleep episodes. The SSA can calculate the amount of activity and the participant’s posture while wearing the MTN-220 device. The “start of time in bed (TIB)” was manually set as “the time when their activity level suddenly decreased, and when they shifted from a standing to a lying posture that was the closest to participants’ self-reported time for going to sleep in the sleep diary.” The “end of TIB” was set in a similar way, but it referred to the time that was the closest to a participant’s self-reported time for waking up in the sleep diary. Using this setup, the other sleep parameters were automatically calculated using SSA. Specifically, manually setting the times for the start and end of TIB and considering both objective and subjective data allowed us to accurately calculate sleep data in the first and main sleep episodes. Hereafter, we provide definitions of the sleep parameters used in this study:

(1) TIB (min): the time at which the posture involved lying.
(2) Sleep latency (SL; min): the interval between changing posture from standing to lying and the start of the sleep episode.
(3) Total sleep time (TST; min): using the start and end of TIB, we calculated the sum of the periods in which participants were judged to have fallen asleep.
(4) Sleep efficiency (SE; %): percentage of TST for TIB.
(5) Wake after sleep onset (WASO; min): the amount of time spent awake during the interval between sleep onset and offset.
(6) Bed-out time (BOT; min): the interval between the last sleep onset and the change in posture from lying to standing.

#### 2.3.4. Burnout

Burnout was defined as a long-term stress reaction resulting from experiencing long-term and repeated stress [23]. We used the verified and reliable 17-item Japanese Burnout Scale, developed in accordance with the Maslach Burnout Inventory, to evaluate participants’ burnout stage [24]. It comprises three subscales: emotional exhaustion, decline in personal accomplishment, and depersonalization. The higher the score, the stronger the symptoms. This scale was implemented at the beginning of the data collection.

#### 2.3.5. Resilience

Psychological resilience was defined as an individual’s ability to adjust positively to adversity [25]. We used the valid and reliable 22-item Japanese Resilience Scale for Nurses [25]. It comprises four subscales: positivity in nursing, interpersonal skills, having an anchor in one’s personal life, and response to novelty; higher scores reflected higher resilience. This scale was implemented at the end of the data collection.

#### 2.3.6. Coping Profile

Workers’ coping profiles greatly affect the process by which job stressors lead to health problems [26]. We used the valid and reliable 18-item Japanese Brief Scales for Coping Profile for workers [26]. It comprises six subscales: active solution, avoidance and suppression, changing mood, changing a point of view, seeking help for a solution, and emotional expression involving others. The higher the score, the more frequent the occurrence of coping behaviors. This scale was implemented at the end of the data collection.

#### 2.3.7. Work Engagement

Work engagement is assumed to be negatively related to burnout, being defined as a positive, fulfilling, work-related state of mind characterized by vigor, dedication, and absorption [27]. We used a valid and reliable 9-item Japanese Utrecht Work Engagement Scale [27]. It comprises three subscales: vigor, dedication, and absorption. Higher scores reflected higher levels of engagement. This scale was implemented at the end of the data collection.

### 2.4. Statistical Analysis

Continuous variables were presented as means and standard deviations, whereas categorical variables were described as frequencies and percentages. The normality of the data was confirmed using the Shapiro–Wilk test. Age-specific comparisons in each data set were analyzed using: (1) one-way analysis of variance and Tukey–Kramer’s honestly significant difference test, (2) Kruskal–Wallis test and Steel–Dwass test, (3) Pearson’s chi-squared test, and (4) Fisher’s exact test.

As a preliminary analysis, the scores at each measurement point for fatigue were analyzed using a linear mixed model. Age, time, and their interactions were set as the fixed effects. Study participants and their wards were considered random effects. The cumulative step count during the night shift was set as a covariate in this study. This was because the cumulative steps during the night shift were related to the change in fatigue before and after the night shift (Supplementary Tables S1 and S2). Moreover, this result was supported by a previous study showing that the cumulative steps during work, shown by wearable devices, were important data that reflected work demands to predict nurses’ fatigue [28]. 

**Figure 2:**
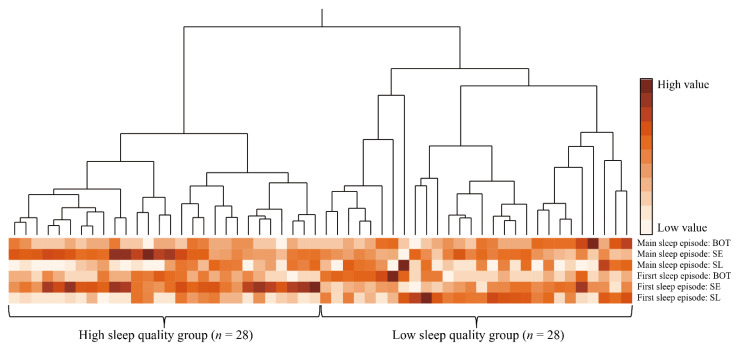
Dendrogram and heatmap of hierarchical cluster analysis with Ward’s method. ***Abbreviations***: BOT, the interval between the last sleep onset and the change in posture from lying to standing; SE, percentage of total sleep time for the time at which the posture involved lying; SL, the interval between changing posture from standing to lying and the start of the sleep episode. ***Notes***: color scales represent value size in each sleep variable; each column represents a participant and each row represents sleep parameter; high sleep quality group identified by cluster analysis had a sleep profile of shorter SL, higher SE, and shorter BOT in both the first and main sleep episodes; low sleep quality group identified by cluster analysis had a sleep profile of longer SL, lower SE, and longer BOT in both the first and main sleep episodes.

After a preliminary analysis exploring associations between variables (Supplementary Tables S1–S4), we analyzed the relationship between recovery from fatigue and sleep episodes after night shifts. An agglomerative hierarchical clustering analysis was used to identify sleep profiles based on multivariate data from SL, SE, and BOT in the first and main sleep episodes after the night shift. This method treats each subject closest in distance as a cluster, and then combines the clusters into consecutively larger clusters based on their proximity [29]. Euclidean distance was used to measure the proximity of variables. Proximity between groups of variables was measured using Ward’s method, which combines clusters by minimizing the sum of the squares of the within-cluster errors. The process of clustering was shown graphically by a dendrogram, which represents the aggregation of subjects into clusters through branching [30]. The optimal number of clusters was determined by referring to the dendrogram (Figure 2) and cubic clustering criterion [31], in addition to clinical interpretability.

Next, the Wilcoxon test, Student’s *t*-test, Pearson’s chi-squared test, and Fisher’s exact test were performed to compare the characteristics between groups identified by cluster analysis. Moreover, comparisons by clusters of changes in fatigue from after the night shift to the morning of the day after the night shift were performed using a linear mixed model. Cluster, age, and their interactions were set as fixed effects. The ward was considered a random effect, and the cumulative steps during the night shift were set as a covariate. In this model, the possibility of confounding by fatigue during the night shift was analyzed in another mixed model adjusted for age, cumulative steps, and ward. We calculated Cohen’s *d*, *φ*, and partial *η*^2^ as the effect size in response to each analysis. Cohen’s *d* was defined to be small (=0.20), medium (=0.50), and large (0.80), and *φ* was defined to be small (=0.10), medium (=0.30), and large (0.50) [32]. Partial *η*^2^ provided benchmarks to define a small (=0.01), medium (=0.06), and large (=0.14) effect [33].

All statistical analyses were conducted using JMP^®^ 16 Pro (SAS Institute Inc., Cary, NC, USA), with *p* < 0.05 denoting statistical significance.

## 3. Results

### 3.1. Participants’ Characteristics

Overall, 62 nurses participated, excluding four that declined to take part in the study. The demographic, work environment, and psychological characteristics of the participants are shown in Table 1. There was no significant difference in the proportion of married nurses and nurses with children among the age groups.

### 3.2. Fatigue by Age Group

Comparisons by age in the time course of fatigue are presented in Supplementary Table S3. Multivariate analysis, adjusting for cumulative steps and wards, showed significant interactions for local pain or dullness and the total score. Overtime changes in subjective fatigue score varied by age, and fatigue in those in their 40 s was significantly lower than that in those in their 20 s. In addition, ward characteristics had no influence on fatigue.

### 3.3. Sleep Episodes and Napping by Age Group

Comparisons by age in napping and sleeping are presented in Supplementary Table S4. In night-shift napping, there were no significant differences between age groups for any of the sleep variables. In the first sleep episode, multiple comparisons by age showed that those in their 40s had a significantly higher SE than those in their 20 s. In the main sleep episode, multiple comparisons by age showed that BOT was significantly shorter among those in their 40 s than among those in their 20 s. There was no effect from the wards.

### 3.4. Relationship between Post-Work Recovery from Fatigue, Sleep Episodes, and Age

Using hierarchical cluster analysis of first and main sleep episodes, participants were classified into two groups, as shown in Figure 2 and Table 2. A total of 56 nurses were included in the analysis because MTN data for six nurses were found to be missing, either with regards to first or main sleep episodes. Cluster A had a sleep profile of shorter SL, higher SE, and shorter BOT in both the first and main sleep episodes; this group was labeled the high sleep quality group (HSQG, *n* = 28). Cluster B had a sleep profile of longer SL, lower SE, and longer BOT in both the first and main sleep episodes; this group was labeled the low sleep quality group (LSQG, *n* = 28). Comparisons between these two groups showed that no statistically significant differences were found in age, start of TIB in the first and main sleep episodes (Table 2), and sleep variables during night-shift napping (Supplementary Table S5). However, the HSQG had a significantly higher percentage of nurses with children (*p* = 0.036, *φ* = 0.28) and a higher score for dedication in work engagement (*p* = 0.043, *d* = 0.39) than the LSQG.

**Figure 3:**
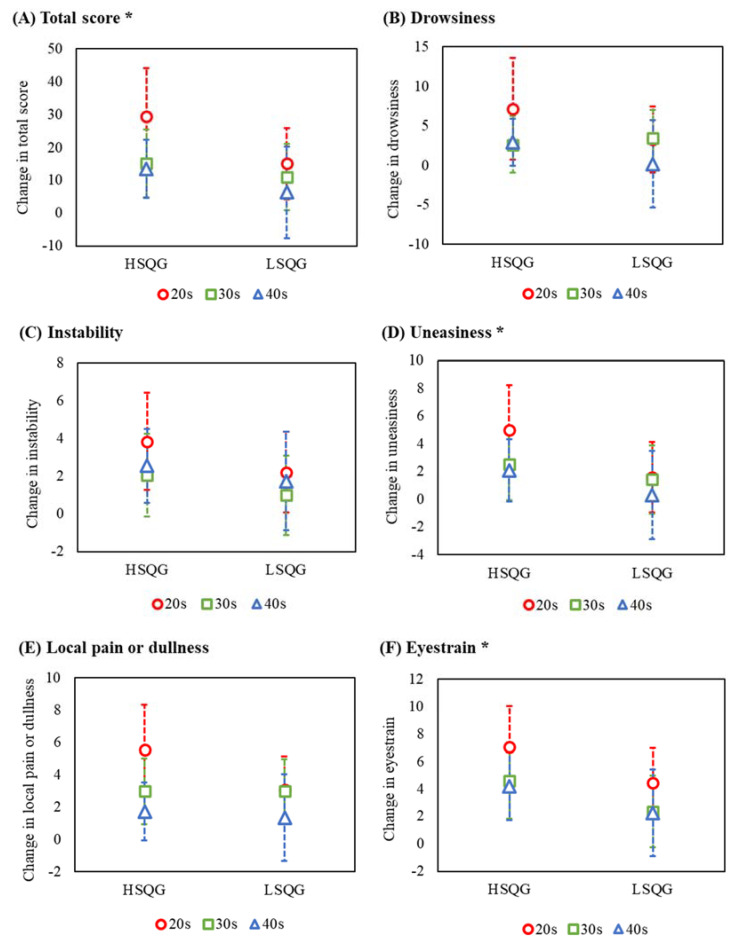
Comparison by cluster of change in fatigue after the night shift to the morning of the next day. ***Abbreviations***: HSQG (*n* = 28), high sleep quality group identified by cluster analysis; LSQG (*n* = 28), low sleep quality group identified by cluster analysis. ***Notes***: change in fatigue (vertical axis), higher scores reflect higher levels of recovery from fatigue; error bar, 95% confidence interval; the circles (nurses in their 20 s), squares (30 s), and triangles (40 s) indicate the least square mean as estimated by the linear mixed model; *, the linear mixed model showed significant main effects of clusters on the total score (**A**), uneasiness (**D**), and eyestrain (**F**); there were no significant differences in the other factors (**B**,**C**,**E**).

The linear mixed model showed significant main effects of clusters on the total score (A) (*F* _[1, 45]_ = 4.41, *p* = 0.041, partial *η*^2^ = 0.09), uneasiness (D) (*F*_[1, 46]_ = 4.45, *p* = 0.041, partial *η*^2^ = 0.09), and eyestrain (F) (*F* _[1, 49]_ = 4.08, *p* = 0.049, partial *η*^2^ = 0.08; Figure 3, Supplementary Table S6). No significant main effects on age ((A) partial *η*^2^ = 0.21, (D) partial *η*^2^ = 0.07, (F) partial *η*^2^ = 0.07) or interaction ((A) partial *η*^2^ = 0.03, (D) partial *η*^2^ = 0.02, (F) partial *η*^2^ = 0.00) were observed. The change in fatigue over time from before to after the night shift was not significantly different between the clusters, indicating that fatigue during the night shift was not a confounding factor in this analysis (total score; main effect of cluster: *F* _[1, 48]_ = 0.14, *p* = 0.707; main effect of time: *F* _[1, 162]_ = 28.14, *p* = < 0.001; interaction: *F* _[1, 162]_ = 1.14, *p* = 0.334).

## 4. Discussion

It is desirable for nurses working long night shifts to promote sufficient recovery from fatigue through appropriate sleep after the night shifts, preventing the transition from acute to chronic fatigue [1,8]. Nonetheless, there is a lack of evidence on the relationship between sleep episodes and recovery from fatigue after night shifts. Thus, our study examined the relationship between recovery from fatigue and sleep episodes after night shifts, considering the effect of age on nurses who worked 16 h night shifts. Taking advantage of the prospective design, we combined multiple sleep parameters in different sleep episodes during non-working time after the night shift to identify the sleep profile of nurses after 16 h night shifts. As for the longitudinal data of fatigue and sleep of nurses working these shifts, “age” had a significant effect, and nurses in their 40s had lower fatigue and better sleep than those in their 20s (Supplementary Tables S3 and S4). The effect size of age on fatigue recovery (total score) was large, showing that age remains an important factor. However, “quality of first and main sleep episodes at home” was associated with recovery from the night shift to the next day, regardless of age. Interestingly, sleep quality after returning home was moderately related to all fatigue factors, despite adjusting for age. Our results are supported by a previous study showing that “sleep quality” was one of the direct effects of acute fatigue among nurses [2]. Our finding emphasizes the need to focus on first and main sleep episodes after night shifts for nurses, suggesting a crucial intervention point for reducing fatigue in nurses working long shifts.

Sleep disturbances among shift nurses are a global problem with prevalence rates ranging from 57.0% to 75.9% [34,35,36,37]. In general, shift workers have a lower quality of sleep during the day following the night shift [38]. We found that poor quality of first and main sleep after night shifts interfered with nurses’ recovery from fatigue. A previous study reported that active and former shift nurses showed more subclinical metabolic abnormalities (higher HbA1c) and altered peripheral clock gene expression than daily work nurses [39]. Moreover, “sleep quality” and “night shift” were independent determinants of higher HbA1c [39]. In other words, low sleep quality among shift nurses is a crucial issue related to not only fatigue recovery but also type 2 diabetes and circadian rhythm disorders [4,40]. To address these, sleep and other factors at the individual’s daily life level should be discussed simultaneously. For instance, the intake of caffeine [41,42], alcohol [43], sweet foods, saturated fat, and cholesterol [44] is associated with sleep quality, which may influence recovery from fatigue. Further, melatonin intake, which regulates circadian rhythms and sleep cycles, can enhance sleep quality [45,46]. Moderate physical activity has also been reported to improve nurses’ sleep duration and quality [4,47]. Although this study focused on the poor quality of first and main sleep after night shifts, the development of comprehensive health strategies (including dietary profile, timing, and physical activity) is needed to induce good sleep after night shifts.

Another important result was that the HSQG had a significantly higher percentage of nurses with children and a higher score for dedication in work engagement than the LSQG. Family (including children) could provide nurses with a prime purpose and personal fulfillment in “working to live” [8], relieving stress and tension, and positively impacting recovery from acute fatigue [1,8]. Furthermore, the presence of children may be a synchronizing factor in their daily rhythms, affecting their sleep. However, family and domestic responsibilities have been reported to increase the home demands of shift-working nurses and interfere with their recovery from fatigue [48]. Our results support former evidence that the presence of children is positive for nurses. In the future, family and domestic responsibilities must also be carefully considered when examining a sleep environment that allows nurses to properly recover from fatigue after long night shifts.

Regarding clinical implications, shift nurses insufficiently focus only on main sleep after night shifts; they should recognize the importance of the first sleep after returning home. For example, nurses could devise the amount and timing of caffeine and alcohol consumption so that they do not interfere with the first sleep after night shifts. Nursing managers should pay attention to educational activities that encourage the importance of sleep after returning home. Moreover, they must strive to reduce overtime after night shifts and avoid climates that enforce it (e.g., by not holding training or meetings after night shifts). Shift scheduling, such as length, rotation speed, and the number of consecutive shifts, would also reduce nurse fatigue [49,50]. However, further research is needed on the sleep environment and individual approaches (including diet and physical activity) that enable high-quality sleep after night shifts. Additionally, more reliable research is needed to clarify the cumulative effect of high-quality sleep after long night shifts in improving shift-work nurses’ recovery levels in the long term and preventing maladaptive chronic fatigue. Such health strategies may be applied to other professions that involve shift work and serve as content for global guidelines.

The strength of our study was the objective measure of nurses’ sleep during the non-working time using accelerometers and the identification of clinically relevant clusters. However, this study has certain limitations. First, the sample size in this study was small because we only observed one night shift per nurse in a general hospital, limiting the generalizability of the results. Additional multicenter, longitudinal cohort studies with larger sample sizes, considering more confounding factors associated with sleep and fatigue, are needed.

## 5. Conclusions

This study examined the relationship between recovery from fatigue and sleep episodes after night shifts while considering the effect of age on nurses who work 16 h night shifts. By identifying the sleep profile of nurses after these shifts, our results suggested that the “quality of first and main sleep episodes at home” was associated with recovery from the night shift to the next day, regardless of age. Therefore, shift nurses should recognize the importance of not only the longest main sleep of the day following the night shift, but also the first sleep after returning home. However, further research is required to understand whether the cumulative effect of high-quality sleep after long night shifts can improve shift work nurses’ recovery levels in the long term and prevent maladaptive chronic fatigue. In addition, a comprehensive health strategy should be developed to induce sleep that promotes recovery from post-night-shift fatigue in nurses.

## Figures and Tables

**Table 1:** Demographic, work environment, and psychological characteristics of participants.

Variable	Total (*n* = 62)	20s (*n* = 20)	30s (*n* = 22)	40s (*n* = 20)	*p*-Value
		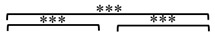			
Age (years): mean (SD)	34.5 (7.6)	26.4 (2.1)	33.2 (2.7)	44.1 (2.5)	<**0.001** ^a^
		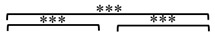			
Years of nursing experience (years): mean (SD)	11.0 (7.1)	4.3 (2.0)	11.1 (3.3)	17.4 (7.3)	<**0.001** ^a^
BMI (kg/m^2^): mean (SD)	21.6 (3.4)	20.8 (2.3)	21.3 (2.6)	22.6 (4.7)	0.616 ^a^
Sex (female): *N* (%)	60 (96.8)	19 (95.0)	21 (95.5)	20 (100.0)	0.999 ^d^
Married (yes): *N* (%)	16 (25.8)	4 (20.0)	8 (36.4)	4 (20.0)	0.371 ^c^
Child rearing (yes): *N* (%)	13 (21.0)	1 (5.0)	6 (27.3)	6 (30.0)	0.096 ^d^
Taking nap before night shift (yes): *N* (%)	18 (29.0)	3 (15.0)	8 (36.4)	7 (35.0)	0.243 ^c^
Napping during night shift (yes): *N* (%)	52 (83.9)	17 (85.0)	16 (72.7)	19 (95.0)	0.197 ^c^
			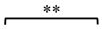		
Resting time during night shift (min): mean (SD)	132.5 (49.3)	135.9 (28.1)	153.0 (62.3)	105.8 (33.1)	**0.002** ^a^
		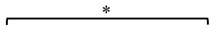			
Cumulative steps during night shift (steps): mean (SD)	9848.3 (2827.5)	11,189.2 (2580.6)	9200.7 (3258.7)	9219.9 (2116.1)	**0.033** ^b^
Frequency of drinking (yes): *N* (%)					
Rarely drinking	22 (35.5)	5 (25.0)	10 (45.4)	7 (35.0)	0.433 ^d^
Drinking every day	3 (4.8)	1 (70.0)	0 (0.0)	2 (10.0)	
Other	37 (59.7)	14 (5.0)	12 (54.6)	11 (55.0)	
Frequency of caffeine intake (yes): *N* (%)					
Rarely drinking	8 (12.9)	3 (15.0)	3 (13.6)	2 (10.0)	**0.002** ^d^
Drinking every day	26 (41.9)	3 (15.0)	8 (36.4)	15 (75.0)	
Other	28 (45.2)	14 (70.0)	11 (50.0)	3 (15.0)	
Burnout: mean (SD)					
Emotional exhaustion	3.5 (1.0)	3.9 (0.7)	3.5 (1.1)	3.2 (1.0)	0.101 ^b^
Decline in personal accomplishment	3.8 (0.6)	3.9 (0.5)	3.8 (0.5)	3.7 (0.7)	0.697 ^a^
Depersonalization	2.3 (1.0)	2.6 (0.9)	2.2 (0.9)	2.1 (0.9)	0.133 ^a^
Resilience: mean (SD)		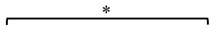			
Total score	71.6 (10.5)	68.0 (13.1)	70.4 (9.4)	76.5 (6.5)	**0.029** ^b^
Positivity in nursing	24.7 (6.0)	24.4 (6.7)	23.3 (6.9)	26.5 (3.7)	0.275 ^a^
		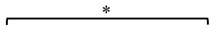			
Interpersonal skills	16.9 (2.8)	15.8 (3.3)	16.8 (2.7)	18.1 (1.9)	**0.025** ^a^
Having an anchor in one’s personal life	20.5 (3.1)	19.3 (3.9)	21.1 (2.6)	21.0 (2.4)	0.105 ^b^
		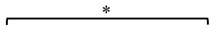			
Response to novelty	9.5 (3.0)	8.5 (3.0)	9.1 (2.5)	11.0 (3.3)	**0.026** ^b^
Coping profile: mean (SD)		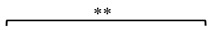			
Active solution	9.2 (1.8)	8.6 (1.1)	9.0 (1.9)	10.3 (1.9)	**0.010** ^a^
Avoidance and suppression	6.5 (2.1)	7.4 (2.3)	6.2 (2.1)	6.0 (1.7)	0.129 ^a^
Changing mood	8.4 (2.4)	9.1 (1.9)	8.2 (2.3)	7.9 (2.8)	0.233 ^b^
Changing a point of view	7.5 (2.0)	7.0 (1.8)	7.5 (2.2)	7.9 (2.1)	0.279 ^a^
Seeking help for a solution	8.9 (2.1)	8.1 (1.9)	9.3 (1.8)	9.2 (2.5)	0.123 ^a^
Emotional expression involving others	4.6 (1.3)	5.0 (1.1)	4.5 (1.5)	4.4 (1.1)	0.183 ^a^
Work engagement: mean (SD)					
Vigor	6.1 (3.2)	5.1 (2.6)	6.5 (3.6)	6.7 (3.0)	0.228 ^b^
Dedication	7.4 (3.3)	6.7 (3.0)	7.7 (3.8)	8.0 (2.9)	0.422 ^a^
Absorption	5.4 (3.0)	4.5 (3.0)	5.3 (3.4)	6.5 (2.5)	0.127 ^a^

***Abbreviations***: BMI, body mass index; SD, standard deviation. ***Notes***: statistically significant values were marked in bold; ^a^ Kruskal–Wallis test and Steel–Dwass test; ^b^ one-way ANOVA and Tukey–Kramer’s honestly significant difference test; ^c^ Pearson’s chi-squared test; ^d^ Fisher’s exact test; *, *p* < 0.05; **, *p* < 0.01; ***, *p* < 0.001.

**Table 2:** Comparison of characteristics between two groups identified by cluster analysis.

Variable	HSQG (*n* = 28)	LSQG (*n* = 28)	Test Statistic	Effect Size	*p*-Value
First sleep episode: cluster mean (SD)					
SL (min)	9.9 (8.2)	30.7 (21.3)	-		-
SE (%)	82.4 (8.8)	63.9 (13.0)	-		-
BOT (min)	7.1 (3.6)	9.4 (7.8)	-		-
Main sleep episode: cluster mean (SD)					
SL (min)	16.9 (14.9)	28.0 (33.8)	-		-
SE (%)	80.4 (10.0)	68.8 (10.1)	-		-
BOT (min)	5.9 (3.2)	12.2 (11.2)	-		-
Age group: *N* (%)					
20s	8 (28.6)	11 (39.3)	1.42	0.16	0.492 ^c^
30s	9 (32.1)	10 (35.7)			
40s	11 (39.3)	7 (25.0)			
Years of nursing experience (years): mean (SD)	10.7 (6.9)	10.4 (7.4)	0.01	0.00	0.922 ^a^
BMI (kg/m^2^): mean (SD)	21.2 (3.3)	21.6 (3.0)	0.17	0.02	0.682 ^a^
Sex (female): *N* (%)	27 (96.4)	27 (96.4)	0.00	0.00	0.999 ^c^
Married (yes): *N* (%)	9 (32.1)	4 (14.3)	2.50	0.21	0.114 ^c^
Child rearing (yes): *N* (%)	8 (28.6)	2 (7.1)	4.38	0.28	**0.036** ^c^
Napping during night shift (yes): *N* (%)	23 (82.1)	23 (82.1)	0.00	0.00	0.999 ^c^
Resting time during night shift (min): mean (SD)	138.8 (62.5)	123.3 (32.3)	1.60	0.21	0.206 ^a^
Cumulative steps during night shift (steps): mean (SD)	9954.5 (2695.1)	9812.1 (3044.8)	0.19	0.05	0.854 ^b^
Frequency of drinking (yes): *N* (%)					
Rarely drinking	10 (35.7)	9 (32.1)	0.50	0.09	0.831 ^d^
Drinking every day	2 (7.2)	1 (3.6)			
Other	16 (57.1)	18 (64.3)			
Frequency of caffeine intake (yes): *N* (%)					
Rarely drinking	5 (17.9)	3 (10.7)	0.88	0.13	0.673 ^d^
Drinking every day	11 (39.3)	10 (35.7)			
Other	12 (42.8)	15 (53.6)			
Start of TIB in first sleep episode: *N* (%)					
AM in the day after the night shift	13 (46.4)	14 (50.0)	0.07	0.04	0.789 ^c^
PM in the day after the night shift	15 (53.6)	14 (50.0)			
Start of TIB in main sleep episode: *N* (%)					.
Before midnight	10 (43.5)	8 (40.0)	0.05	0.03	0.818 ^c^
After midnight	13 (56.5)	12 (60.0)			
Work engagement: Mean (SD)					
Vigor	6.6 (3.6)	5.6 (2.7)	1.14	0.31	0.261 ^b^
Dedication	8.1 (3.8)	6.8 (2.8)	4.11	0.39	**0.043** ^a^
Absorption	5.8 (3.3)	4.9 (2.7)	1.49	0.30	0.222 ^a^

***Abbreviations***: BMI, body mass index; BOT, the interval between the last sleep onset and the change in posture from lying to standing; HSQG, high sleep quality group identified by cluster analysis; LSQG, low sleep quality group identified by cluster analysis; SD, standard deviation; SE, percentage of total sleep time for TIB; SL, the interval between changing posture from standing to lying and the start of the sleep episode; TIB, the time at which the posture involved lying. ***Notes***: statistically significant values were marked in bold; ^a^ Wilcoxon test; ^b^ Student’s *t*-test; ^c^ Pearson’s chi-squared test; ^d^ Fisher’s exact test; ^e^ there were missing data.

## Data Availability

Data sharing not applicable.

## Supplementary Materials

The following supporting information can be downloaded at: https://www.mdpi.com/article/10.3390/healthcare10061089/s1, Table S1: Univariate analyses of changes in fatigue before and after the night shift and demographic or work environment characteristics of participants; Table S2: Univariate analyses of changes in fatigue before and after the night shift and sleep parameters; Table S3: Comparisons by age in the time courses of fatigue; Table S4: Comparisons by age in sleep parameters; Table S5: Comparison of napping during night shift between two groups identified by cluster analysis; Table S6: The results of the linear mixed model for relationship between post-work recovery from fatigue, sleep episodes, and age.
