# Supplementary material for: Post-Work Recovery from Fatigue and Sleep Episodes among Nurses Who Are Engaged in 16-Hour Night Shifts: A Prospective Observational Study
Source: Healthcare (Basel). 2022 Jun 11;10(6):1089. doi: 10.3390/healthcare10061089 (PMC9222225; doi:10.3390/healthcare10061089)
Supplement: Supplementary file 1 [file healthcare-10-01089-s001.zip › healthcare-1721967-supplementary/Healthcare_Supplementary Table S1.pdf]

Supplementary Table S1.
Univariate analyses of changes in fatigue before and after the night shift and demographic or work environment characteristics of participants.

Variable (n = 62)	Drowsiness	Instability	Uneasiness	Local pain or dullness	Eyestrain	Total score
Age [years] [a]	–0.067	0.048	–0.028	–0.210	–0.098	–0.098
Years of nursing experience [years] [a]	–0.033	–0.053	–146	–0.171	–0.083	–0.123
BMI [kg/m^2] [a]	–0.116	–0.056	–0.010	–0.226	–0.088	–0.132
Resting time during night shift [min] [a]	–0.039	–0.031	–0.092	–0.043	–0.098	–0.081
Napping time during night shift [min] [a]	–0.027	–0.183	–0.045	–0.024	–0.188	–0.113
**Cumulative steps during night shift [steps]** [a]	0.156	0.190	0.233	**0.374** **	**0.329** **	**0.330** **
Sex [b]						
Female (n = 60)	4.1 (4.7)	–0.1 (2.9)	2.1 (3.6)	3.2 (3.5)	3.8 (3.9)	13.2 (14.3)
Male (n = 2)	3.0 (0.0)	–2.5 (3.5)	0.5 (0.7)	–0.5 (0.7)	–0.5 (0.7)	0.0 (4.3)
Married [b]						
Yes (n = 16)	5.4 (4.5)	–0.5 (3.0)	2.6 (3.9)	3.7 (3.3)	4.6 (4.1)	15.9 (14.8)
No (n = 46)	3.6 (4.6)	–0.1 (2.9)	1.9 (3.4)	2.9 (3.6)	3.4 (3.9)	11.6 (14.1)
Child rearing [b]						
Yes (n = 13)	5.2 (4.9)	0.7 (3.0)	3.1 (4.5)	3.3 (3.8)	4.4 (3.8)	16.7 (16.8)
No (n = 49)	3.8 (4.6)	–0.4 (2.8)	1.8 (3.3)	3.0 (3.5)	3.5 (4.0)	11.7 (13.6)
Taking nap before night shift [b]						
Yes (n = 18)	4.4 (5.0)	0.1 ((2.8)	2.2 (3.6)	3.4 (4.1)	3.6 (3.5)	13.8 (16.0)
No (n = 44)	4.0 (4.5)	–0.3 (2.9)	2.0 (3.6)	3.0 (3.3)	3.7 (4.1)	12.3 (13.7)
Napping during night shift [b]						
Yes (n = 52)	4.1 (4.7)	–0.2 (2.9)	2.0 (3.7)	3.2 (3.6)	3.7 (4.0)	12.8 (14.8)
No (n =10)	4.1 (4.5)	–0.4 (2.6)	2.5 (2.9)	2.6 (2.9)	3.8 (3.7)	12.6 (11.8)
Frequency of drinking [b]						
Rarely drinking (n =22)	4.8 (5.0)	–0.0 (3.3)	2.5 (3.9)	3.6 (2.9)	3.4 (3.1)	14.4 (14.6)
Drinking every day (n = 3)	5.3 (4.7)	–1.0 (2.0)	3.7 (2.9)	2.7 (3.9)	6.7 (3.5)	18.3 (4.5)
Other (n = 37)	3.6 (4.5)	–0.3 (2.7)	1.7 (3.4)	3.7 (2.5)	3.6 (4.4)	11.3 (14.7)
Frequency of caffeine intake [b]						
Rarely drinking (n = 8)	3.9 (5.8)	–0.1 (4.1)	1.9 (3.5)	3.1 (4.1)	6.0 (4.4)	14.8 (15.9)
Drinking every day (n = 26)	4.7 (4.6)	0.2 (2.2)	2.3 (3.3)	3.4 (3.6)	3.4 (3.2)	14.0 (13.3)
Other (n = 28)	3.6 (4.4)	–0.6 (3.1)	1.9 (3.9)	2.8 (3.3)	3.3 (4.3)	11.0 (15.1)
Ward [b]						
A (n = 22)	2.1 (4.3)	–0.4 (3.1))	1.3 (4.3)	2.5 (3.9)	3.9 (5.0)	9.5 (16.7)
B (n = 8)	5.3 (3.3)	0.0 (2.8)	2.3 (2.9)	3.1 (3.1)	3.5 (3.1)	14.1 (12.2)
C (n = 6)	7.3 (8.8)	1.3 (3.6)	5.5 (5.0)	5.2 (4.6)	4.5 (2.3)	23.8 (22.3)
D (n = 9)	4.7 (3.1)	0.3 (3.3)	2.9 (2.6)	2.3 (3.0)	4.6 (4.3)	14.8 (10.7)
E (n = 5)	3.4 (5.4)	–1.2 (0.8)	0.6 (2.9)	3.2 (3.3)	1.4 (3.0)	7.4 (12.0)
F (n = 12)	5.2 (3.1)	–0.8 (2.6)	1.7 (1.2)	3.7 (3.0)	3.3 (3.1)	13.0 (6.5)

Abbreviations: BMI, body mass index; SD, standard deviation.

Notes: Statistically significant values were marked in bold; a, Continuous variables: Correlation coefficients between fatigue scores and variables are shown in table; b, Categorical variables: Mean (SD) of changes in fatigue from before to after the night shift in each category is in shown in table; **, *P* < .01.
