# Supplementary material for: Post-Work Recovery from Fatigue and Sleep Episodes among Nurses Who Are Engaged in 16-Hour Night Shifts: A Prospective Observational Study
Source: Healthcare (Basel). 2022 Jun 11;10(6):1089. doi: 10.3390/healthcare10061089 (PMC9222225; doi:10.3390/healthcare10061089)
Supplement: Supplementary file 1 [file healthcare-10-01089-s001.zip › healthcare-1721967-supplementary/Healthcare_Supplementary Table S2.pdf]

Supplementary Table S2.
Univariate analyses of changes in fatigue before and after the night shift and sleep parameters.

Variable	Drowsiness	Instability	Uneasiness	Local pain or dullness	Eyestrain	Total score
Night-shift napping (n = 52) [a]						
Start of TIB [hh:mm]	–0.001	–0.001	–0.027	0.017	0.057	0.010
TIB [min]	0.082	–0.166	–0.083	0.158	–0.228	–0.052
SL [min] [c]	0.163	–0.053	0.164	0.268	**0.284 ***	0.224
TST [min]	0.018	–0.132	–0.127	0.008	**–0.341 ***	–0.144
SE [%] [c]	–0.044	0.060	–0.047	–0.162	–0.208	–0.110
WASO [min]	0.035	0.007	–0.001	0.135	0.005	0.046
BOT [min] [c]	0.017	–0.273	–0.178	0.198	–0.069	–0.064
End of TIB [hh:mm]	0.023	–0.067	–0.052	0.079	–0.015	–0.004
First sleep episode (n = 56) [a]						
Start of TIB [hh:mm]	–0.243	–0.056	**–0.272 ***	–0.175	–0.203	–0.259
TIB [min]	0.131	0.202	0.104	0.055	0.087	0.147
SL [min] [c]	–0.044	0.069	–0.040	0.059	–0.058	–0.012
TST [min]	0.121	0.222	0.101	0.041	0.069	0.139
SE [%] [c]	0.090	0.140	0.138	0.033	0.102	0.129
WASO [min]	0.105	0.004	0.074	0.034	0.117	0.095
BOT [min] [c]	0.064	–0.027	0.033	0.000	0.051	0.038
End of TIB [hh:mm]	–0.097	0.115	–0.144	–0.101	–0.099	–0.098
Main sleep episode (n = 61) [a]						
Start of TIB [hh:mm]	0.113	–0.149	0.119	**0.316 ***	0.115	0.146
TIB [min]	–0.097	0.229	0.024	0.046	–0.019	0.027
SL [min] [c]	0.043	0.086	0.029	0.231	–0.099	0.068
TST [min]	–0.127	0.056	–0.045	0.044	0.035	–0.021
SE [%] [c]	–0.138	**–0.260 ***	–0.133	–0.024	0.076	–0.116
WASO [min]	0.025	**0.311 ***	0.109	–0.076	–0.049	0.066
BOT [min] [c]	–0.068	0.044	0.034	–0.025	–0.063	–0.028
End of TIB [hh:mm]	0.019	0.059	0.126	**0.318 ***	0.086	0.152

***Abbreviations*:** BOT, the interval between the last sleep onset and the change in posture from lying to standing; SD, standard deviation; SE, percentage of TST for TIB; SL, the interval between changing the posture from standing to lying and the start of the sleep episode; TIB, the time at which the posture involved lying; TST, using the start and end of TIB, we calculated the sum of periods in which participants were judged to have fallen asleep; WASO, the amount of min spent awake during the interval between sleep onset and offset.

***Notes*:** Statistically significant values were marked in bold; a, Continuous variables: Correlation coefficients between fatigue scores and variables are shown in table; *, $P < .05$.
