# Supplementary material for: Post-Work Recovery from Fatigue and Sleep Episodes among Nurses Who Are Engaged in 16-Hour Night Shifts: A Prospective Observational Study
Source: Healthcare (Basel). 2022 Jun 11;10(6):1089. doi: 10.3390/healthcare10061089 (PMC9222225; doi:10.3390/healthcare10061089)
Supplement: Supplementary file 1 [file healthcare-10-01089-s001.zip › healthcare-1721967-supplementary/Healthcare_Supplementary Table S3.pdf]

Supplementary Table S3. Comparisons by age in the time courses of fatigue.

Variable: Mean (SD)	Time					Main effect: Age		Main effect: Time		Interaction: Age × Time	
	Before night shift [c]	Before napping	After napping	After night work	Morning in next day after night work	*F(df)*	*P* [a]	*F(df)*	*P* [a]	*F(df)*	*P* [a]
Drowsiness											
20s (n = 20)	12.4 (5.4)	15.1 (5.7) †	15.4 (5.4) †	16.2 (5.6) ‡	11.3 (5.2)						
30s (n = 22)	10.2 (4.8) **	14.4 (4.9) ‡	16.1 (5.6) ‡	15.5 (6.3) ‡ *	11.9 (4.7)	3.35 (2, 17)	.060	28.76 (4, 236)	**< .001**	1.38 (8, 236)	.204
40s (n = 20)	7.9 (2.9)	12.1 (4.6) ‡	13.1 (4.6) ‡	11.0 (4.5) ‡	9.2 (4.1)						
P-value [b]	**.010**	.154	.168	**.008**	.148						
Instability											
20s (n = 20)	11.4 (5.4)	11.4 (5.3)	11.7 (6.5)	10.9 (5.9)	7.3 (4.4) ‡						
30s (n = 22)	9.4 (3.8)	9.3 (4.3)	10.2 (5.1)	9.2 (4.5)	7.6 (3.8) †	1.22 (2, 51.1)	.305	19.93 (4, 236)	**< .001**	1.52 (8, 236)	.151
40s (n = 20)	8.0 (4.0)	8.1 (4.0)	8.3 (3.6)	8.1 (4.5)	6.2 (1.8) ‡						
P-value [b]	.060	.074	.124	.226	.416						
Uneasiness											
20s (n = 20)	10.2 (3.7)	11.5 (3.9)	12.4 (4.6) †	12.2 (5.6)	8.6 (4.4)						
30s (n = 22)	8.2 (3.2) *	9.5 (4.1) *	11.7 (5.3) ‡ *	10.7 (4.9) ‡	8.6 (4.0)	3.07 (2, 293.0)	**.048**	18.15 (4, 294)	**< .001**	1.91 (8, 294)	.060
40s (n = 20)	6.9 (2.4)	8.0 (2.9)	8.6 (2.6) †	8.5 (4.3) †	7.5 (2.6)						
P-value [b]	**.006**	**.015**	**.018**	.066	.555						
Local pain or dullness											
20s (n = 20)	10.0 (3.6)	12.6 (4.6) ‡	12.1 (5.1) †	14.1 (6.2) ‡	9.5 (4.8)						
30s (n = 22)	9.3 (4.4)	11.8 (4.6) ‡	12.0 (5.5) ‡	12.8 (5.6) ‡	9.9 (4.8)	0.86 (2, 47)	.432	34.36 (4, 236)	**< .001**	2.59 (8, 236)	**.010**
40s (n = 20)	8.6 (3.8)	9.8 (4.0) †	10.0 (4.0) ‡	10.3 (4.4) ‡	8.9 (3.9)						
P-value [b]	.543	.127	.292	.091	.751						
Eyestrain											
20s (n = 20)	10.0 (4.2)	13.3 (4.2) ‡	14.2 (5.5) ‡	14.4 (5.5) ‡	8.5 (5.1)						
30s (n = 22)	8.9 (3.4)	11.9 (4.7) ‡	13.5 (5.5) ‡ *	12.5 (5.5) ‡	8.9 (3.7)	0.16 (2, 39)	.217	40.12 (4, 236)	**< .001**	1.79 (8, 236)	.078
40s (n = 20)	7.6 (3.9)	10.7 (4.9) ‡	9.9 (5.0) ‡	10.6 (4.6) ‡	7.6 (3.2)						
P-value [b]	.145	.225	**.028**	.076	.551						
Total score											
20s (n = 20)	53.9 (19.2)	63.8 (20.8) †	65.7 (25.6) ‡	67.7 (26.0) ‡	45.2 (21.6) †						
30s (n = 22)	46.0 (17.5) *	56.9 (20.6) ‡	63.5 (24.9) ‡	60.7 (25.0) ‡ *	47.0 (18.7)	2.03 (2, 16)	.163	39.80 (4, 236)	**< .001**	2.01 (8, 236)	**.046**
40s (n = 20)	38.9 (14.6)	48.7 (16.6) ‡	49.8 (16.9) ‡	48.4 (18.6) ‡	39.3 (13.0)						
P-value [b]	**.028**	.056	.064	**.037**	.367						

Abbreviations: df, degree of freedom; SD, standard deviation. ***Notes***: Statistically significant values were marked in bold; a, Linear mixed model; b, One-way ANOVA and Tukey–Kramer's honestly significant difference (HSD) test (*, $P < .05$. **, $P < .01$); c, Dunnett's test (before night work = control; †, $P < .05$, ‡, $P < .01$).
