# Supplementary material for: Post-Work Recovery from Fatigue and Sleep Episodes among Nurses Who Are Engaged in 16-Hour Night Shifts: A Prospective Observational Study
Source: Healthcare (Basel). 2022 Jun 11;10(6):1089. doi: 10.3390/healthcare10061089 (PMC9222225; doi:10.3390/healthcare10061089)
Supplement: Supplementary file 1 [file healthcare-10-01089-s001.zip › healthcare-1721967-supplementary/Healthcare_Supplementary Table S4.pdf]

Supplementary Table S4. Comparisons by age in sleep parameters.

Variable	Total	20s	30s	40s	*P*-value
Night-shift napping: Mean (SD)	n = 52	n = 17	n = 16	n = 19	
Start of TIB [hh:mm]	26:09 (1:39)	25:42 (1:38)	26:05 (1:23)	26:37 (1:45)	.432 [a]
TIB [min]	91.6 (31.2)	91.8 (25.5)	103.9 (32.8)	80.5 (32.5)	.105 [a]
SL [min]	10.2 (10.1)	10.7 (10.7)	9.3 (8.7)	10.4 (11.3)	.844 [b]
TST [min]	70.6 (28.8)	69.2 (20.6)	81.3 (28.7)	62.6 (34.1)	.180 [a]
SE [%]	76.9 (15.6)	75.5 (12.9)	79.3 (13.3)	75.9 (19.3)	.684 [b]
WASO [min]	6.6 (10.0)	6.6 (6.5)	9.7 (15.8)	3.9 (4.7)	.357 [b]
BOT [min]	4.2 (3.3)	5.3 (4.7)	3.5 (2.1)	3.6 (2.1)	.635 [b]
End of TIB [hh:mm]	27:40 (1:34)	27:15 (1:34)	27:45 (1:32)	27:57 (1:33)	.329 [b]
First sleep episode: Mean (SD)	n = 56	n = 19	n = 19	n = 18	
Start of TIB [hh:mm]	12:38 (1:55)	12:38 (1:41)	11:55 (1:17)	13:25 (2:22)	.062 [a]
TIB [min]	252.4 (112.8)	266.8 (130.0)	239.6 (110.7)	250.8 (99.2)	.762 [b]
SL [min]	20.3 (19.2)	20.3 (16.9)	22.9 (22.8)	17.4 (17.9)	.678 [b]
TST [min]	189.4 (96.6)	188.9 (101.2)	181.3 (86.5)	198.4 (106.2)	.834 [b]
		* (20s vs 40s)			
SE [%]	73.2 (14.5)	68.3 (12.0)	75.0 (12.1)	76.3 (13.1)	**.032** [b]
WASO [min]	34.5 (34.0)	46.9 (45.0)	27.9 (24.0)	28.4 (26.8)	.586 [b]
BOT [min]	8.2 (6.2)	10.6 (8.8)	7.5 (3.8)	6.4 (3.7)	.260 [b]
End of TIB [hh:mm]	16:51 (2:19)	17:05 (2:05)	15:54 (1:56)	17:35 (2:34)	.077 [a]
Main sleep episode: Mean (SD)	n = 61	n = 20	n = 21	n = 20	
Start of TIB [hh:mm]	23:49 (3:44)	25:08 (2:12)	22:42 (5:13)	23:43 (2:19)	.069 [a]
TIB [min]	542.4 (124.3)	535.2 (122.6)	574.0 (133.1)	516.5 (115.2)	.323 [a]
SL [min]	21.5 (25.8)	22.9 (34.6)	20.3 (16.4)	21.5 (24.7)	.614 [b]
TST [min]	405.1 (111.1)	386.1 (114.1)	435.7 (107.6)	392.0 (110.5)	.531 [b]
SE [%]	74.6 (11.8)	71.7 (13.5)	76.6 (11.3)	75.3 (9.7)	.395 [a]
WASO [min]	105.7 (63.2)	111.5 (59.3)	108.7 (78.8)	96.9 (49.1)	.746 [a]
		* (20s vs 40s)			
BOT [min]	10.0 (10.6)	14.7 (14.3)	9.3 (9.2)	6.1 (4.3)	**.036** [b]
End of TIB [hh:mm]	9:15 (2:28)	10:03 (2:13)	9:21 (2:09)	8:19 (2:42)	.089 [a]

***Abbreviations*:** BOT, the interval between the last sleep onset and the change in posture from lying to standing; SD, standard deviation; SE, percentage of TST for TIB; SL, the interval between changing the posture from standing to lying and the start of the sleep episode; TIB, the time at which the posture involved lying; TST, using the start and end of TIB, we calculated the sum of periods in which participants were judged to have fallen asleep; WASO, the amount of min spent awake during the interval between sleep onset and offset.

***Notes*:** Statistically significant values were marked in bold; a, One-way ANOVA and Tukey–Kramer's honestly significant difference (HSD) test. b, Kruskal-Wallis test and Steel-Dwass test; *, $P < .05$.
