# Supplementary material for: Post-Work Recovery from Fatigue and Sleep Episodes among Nurses Who Are Engaged in 16-Hour Night Shifts: A Prospective Observational Study
Source: Healthcare (Basel). 2022 Jun 11;10(6):1089. doi: 10.3390/healthcare10061089 (PMC9222225; doi:10.3390/healthcare10061089)
Supplement: Supplementary file 1 [file healthcare-10-01089-s001.zip › healthcare-1721967-supplementary/Healthcare_Supplementary Table S5.pdf]

Supplementary Table S5.

Comparison of napping during night shift between two groups identified by cluster analysis.

Variable	HSQG (n = 28)	LSQG (n = 28)	Effect size	*P*-value
Napping during night shift [Yes]: N (%)	23 (82.1)	23 (82.1)	0.00	.999 [c]
Resting time during night shift [min]: Mean (SD)	138.8 (62.5)	123.3 (32.3)	0.31	.206 [a]
TIB [min]: Mean (SD)	85.5 (32.5)	92.3 (23.4)	0.24	.428 [b]
SL [min]: Mean (SD)	10.1 (11.1)	8.4 (7.8)	0.18	.872 [a]
TST [min]: Mean (SD)	67.1 (29.9)	73.5 (20.8)	0.25	.417 [b]
SE [%]: Mean (SD)	78.5 (15.9)	78.9 (11.7)	0.03	.716 [a]
WASO [min]: Mean (SD)	4.4 (5.6)	6.2 (7.3)	0.28	.445 [a]
BOT [min]: Mean (SD)	3.8 (4.0)	4.3 (2.8)	0.15	.416 [a]

Abbreviations: BOT, the interval between the last sleep onset and the change in posture from lying to standing; HSQG, high sleep quality group identified by cluster analysis; LSQG, low sleep quality group identified by cluster analysis; SD, standard deviation; SE, percentage of TST for TIB; SL, the interval between changing the posture from standing to lying and the start of the sleep episode; TIB, the time at which the posture involved lying; TST, using the start and end of TIB, we calculated the sum of periods in which participants were judged to have fallen asleep; WASO, the amount of min spent awake during the interval between sleep onset and offset.

Notes: a, Wilcoxon test; b, Student's t-test; c; Pearson's chi-squared test.
