# Supplementary material for: Post-Work Recovery from Fatigue and Sleep Episodes among Nurses Who Are Engaged in 16-Hour Night Shifts: A Prospective Observational Study
Source: Healthcare (Basel). 2022 Jun 11;10(6):1089. doi: 10.3390/healthcare10061089 (PMC9222225; doi:10.3390/healthcare10061089)
Supplement: Supplementary file 1 [file healthcare-10-01089-s001.zip › healthcare-1721967-supplementary/Healthcare_Supplementary Table S6.pdf]

Supplementary Table S6.

The results of the linear mixed model for relationship between post-work recovery from fatigue, sleep episodes, and age.

	P-value	*F*-value (*df*)	Partial η^2 value
[A] Total score			
Main effect of cluster	**.041**	4.41 (1, 45)	0.09
Main effect of age	.108	2.51 (2, 19)	0.21
Interaction between cluster and age	.605	0.51 (2, 37)	0.03
[B] Drowsiness			
Main effect of cluster	.164	2.00 (1, 44)	0.04
Main effect of age	.216	1.77 (2, 11)	0.24
Interaction between cluster and age	.407	0.93 (2, 26)	0.07
[C] Instability			
Main effect of cluster	.172	1.93 (1, 47)	0.04
Main effect of age	.454	0.80 (2, 41)	0.04
Interaction between cluster and age	.929	0.07 (2, 48)	0.00
[D] Uneasiness			
Main effect of cluster	**.041**	4.45 (1, 46)	0.09
Main effect of age	.313	1.21 (2, 30)	0.07
Interaction between cluster and age	.632	0.46 (2, 45)	0.02
[E] Local pain or dullness			
Main effect of cluster	.264	1.28 (1, 47)	0.03
Main effect of age	.064	3.17 (2, 20)	0.24
Interaction between cluster and age	.445	0.83 (2, 36)	0.04
[F] Eyestrain			
Main effect of cluster	**.049**	4.08 (1, 49)	0.08
Main effect of age	.162	1.89 (2, 49)	0.07
Interaction between cluster and age	.972	0.03 (2, 49)	0.00

Notes: This mixed model was constructed with the following setup; Cluster (high sleep quality group, low sleep quality group), age (20s, 30s, 40s), and their interactions were set as fixed effects; The ward was considered a random effect, and the cumulative step during the night shift was set as a covariate. Statistically significant (*P*-values) were marked in bold.
